# Supplementary material for: Accuracy of online symptom checkers and the potential impact on service utilisation
Source: PLoS One. 2021 Jul 15;16(7):e0254088. doi: 10.1371/journal.pone.0254088 (PMC8282353; doi:10.1371/journal.pone.0254088)
Supplement: S1 Appendix — (DOCX) [file pone.0254088.s001.docx]

***S1 Appendix***

This appendix includes more details on study sample search criteria, inclusions and exclusions of symptom checker providers, the standardised clinical vignettes that were used, diagnosis and triage accuracy for each symptom checker.

**S1 Table. *Search Terms.*** Contain the search terms to generate the list of symptom checker inclusions and exclusions lists

**S2 Table. *Symptom checkers included in the study*.** Contain further details of the symptom checkers that were included in the study; including system description, registered country and founding year, as well as estimated monthly visits

**S3 Table. Excluded Symptom Checkers.** Contain the symptom checkers that were assessed but that met the exclusion criteria with a description of the reason for exclusion

**S4 Table. Clinical Vignettes.** Contain the clinical vignettes used to test each of the symptom checkers. Each vignette provided the age, gender, symptoms, and correct diagnosis for a given condition. This table also notes where we added additional symptoms if the symptom checkers asked for them. The “simplified” symptoms were those inputted into each symptom checker and the recommended triage advice was the decisions the clinical team supporting the study agreed upon from NICE Clinical Knowledge Summaries.

**S5 Table. Additional data table.** All Vignettes

**S6 Table. Additional data table.** Emergent Care Vignettes

**S7** **Table. Additional data table**. Urgent Care Vignettes

**S8 Table. Additional data table**. Non-Urgent Care Vignettes

**S9** **Table. Additional data table**. Self- Care Vignettes

***S1 | Search Terms***

| **Search Provider** | **Search Type** | **Search Terms** |
| --- | --- | --- |
| Google web search | Online Tools | “Symptom Checker”  “Symptom Assessment”  “AI Symptom Assessment”  “AI Symptom Checker” |
| Google Play on a Samsung Device | Apps | “Symptom Checker App”  “Symptom Assessment App” |
| App Store on an Apple Device | Apps | “Symptom Checker App” “Symptom Assessment App” |

***S2 | Symptom checkers included in the study***

| **Provider** | **Overview ^1^** | **Founded ^2^** | **Funding ^2^** | **Registered ^2^** | **Monthly Visits ^2^** | **Medical Device Registration ^3^** | **Disclaimer ^1^** |
| --- | --- | --- | --- | --- | --- | --- | --- |
| Ada Health | Ada is a global health company founded by doctors, scientists, and industry pioneers to create new possibilities for personal health. Ada’s core system connects medical knowledge with intelligent technology to help all people actively manage their health and medical professionals to deliver effective care. Ada is proud to collaborate with leading health systems and global non-profit organizations to carry out this vision. The #1 medical app for 130 countries, 10 million assessments have been completed since its global launch in 2016. | 2011 | $69.3m | Germany | 375,630 | Class 1 Medical device | Yes |
| Ask NHS – Sensely | Sensely’s avatar and chatbot-based platforms assist insurance plan members and patients with the insurance services and healthcare resources they need, when they need it. With offices in London and San Francisco, Sensely’s global teams provide virtual assistant solutions to insurance companies, pharmaceutical clients, and hospital systems worldwide | 2013 | $26.8m | USA | 2,838 |  | Yes |
| Babylon Health | Babylon is a digital health service provider that combines AI technology with the medical expertise of humans. Babylon delivers full access to healthcare, including personalised health assessments, treatment advice and face-to-face appointments with a doctor 24/7.  Babylon’s mission is to put an accessible and affordable health service in the hands of every person on earth. With the Babylon app, you can talk to a GP within minutes via phone or video call, ask simple medical questions via their text service and monitor your health with their comprehensive tracking system. The Babylon app is everything you need to proactively manage your healthcare.  Babylon has partnerships will Tencent, TELUS and Samsung. They have AI licensing deals with Prudential Asia and BUPA. They also offer end-to-end clinical services in the UK, Rwanda and Canada. | 2013 | $635.3m | UK | 93,332 | Class 1 | Yes |
| Buoy Health | Buoy provides a personalized all-in-one platform to help guide consumers back to health. Whether it's chatting about symptoms with Buoy Assistant to get help in the moment of sickness or researching benefits through Buoy Dashboard, Buoy is available 24/7 to help make self-diagnosis and navigating the healthcare system simple and easy. For employers, Buoy can be custom configured to surface benefits information and wellness programs, guide employees to in-network providers, and fully integrate with other health portals — providing a single, simple destination for employee wellbeing. | 2014 | $29m | USA | 461,310 |  | Yes |
| CAIRDR - Clinova | Clinova is a UK based, global, consumer healthcare company, with the aim of identifying, developing and marketing innovative and cost-effective healthcare products and digital healthcare solutions. The company’s HQ is close to Old Street in London.  Clinova’s products can be found in the UK at Boots, Tesco, Holland and Barrett, Lloyds Pharmacy, Wiggle, Ocado, and Amazon Prime. Furthermore, you can purchase the products in 20 international markets including the UAE, Singapore, Honk Kong, Japan, Chile, USA, Canada, Ireland, Italy, Portugal, Lebanon, and Oman. | 2006 |  | UK |  |  | Yes |
| Doctorlink | Doctorlink is the UK’s leading health and symptom assessment platform, providing 24/7 access to healthcare for over 10 million NHS patients. Through its clinical decision algorithm, Doctorlink is transforming the industry, enabling payers and providers to save cost, improve efficiencies, and increase the accessibility of healthcare. The platform helps people engage in their health and wellbeing, guiding them to the right care, leading to better outcomes and driving prevention.  Founded by Eight Roads, a global proprietary investment firm backed by Fidelity, and headquartered in the UK with offices in the US, Doctorlink has over 125 employees globally. It provides fully customisable and scalable SaaS products for the world’s largest healthcare organisations including AXA, BUPA, Kaiser Permanente and Web MD, and has provided 60 million health assessments to date. Doctorlink is proud to be the leading provider of online Symptom Assessment to the NHS, covering 1,350 GP practices and available to 10 million patients in England. Doctorlink has recently been selected for the GP IT Futures Framework.  Doctorlink’s algorithms are medico-legal compliant and indemnified with rigorous clinical governance and licensed independent peer review. Built by a team of healthcare experts and technology visionaries, the algorithms combine Bayesian logic to ensure robust clinical safety and AI learning to drive continuous improvement methodology. | 2016 | £20m | UK | 57,826 | Class 1 Medical device | Yes |
| Infermedica | Infermedica tackles the problem of unnecessary medical care and waste, providing insurance companies, hospitals, and health systems with a set of advanced preliminary diagnosis and triage tools.  Founded in 2012 by a team of engineers, data scientists, and physicians, Infermedica develops its own AI technology to collect intake, check symptoms, and guide patients to the right care. The technology can also detect emergencies and support healthcare professionals by giving them early access to health information and recommendations.  Today, over 30 companies worldwide use Infermedica to increase their efficiency, improve patient flow, and reduce costs. Customers include, among others, European-based insurance giant Allianz, Taipei Medical University Hospital, and Everyday Health. | 2012 | $3.7m | Poland | 9,921 | Class 1 | Yes |
| Isabel Healthcare | Isabel Healthcare provides a diagnosis decision support tool that doctors rely on for their own second opinion on patient care. They have developed various apps related to medical for Android and iOS users like Isabel. | 1999 |  | USA | 58,253 |  | Yes |
| Mediktor | Mediktor is the most advanced and accurate expert system for pre-diagnoses, triage and decision-making support.  The first artificial intelligent system clinically validated that is transforming healthcare for everyone.  A technology that finally makes it possible to reduce access barriers to healthcare:  - Patients can make better decisions from the first symptoms  - And the healthcare system can organize its demand and optimize resources to offer a better medical coverage. | 2011 | €3m | Spain | 10,405 | Class 1 | Yes |
| NHS 111 | 111 online is a fast and convenient alternative to the 111 phone service and provides an option for people who want to access 111 digitally to;   - find out how to get the right healthcare in their area, including whether they need to see a GP or seek urgent care - get advice on self-care - in most areas, get a call back from a nurse, doctor or other trained health professional if they need it   It is one of several digital NHS services that are empowering people to manage their own health and care. It has been tested and made increasingly available across England since March 2017.  In 2019 we will continue to work with local services, to make sure that the whole country can use the online 111 service to find the right care. This will include exploring how 111 online can be linked to primary care services. | 2013 |  | UK |  |  | Yes |
| Web.MD | WebMD Health is an online platform that provides health information services for patients, physicians, and health care professionals.  The WebMD content staff specializes in journalism, content creation, community services, expert commentary, and medical review to give its users a variety of ways to find what they are looking for. It is dictated to helping people find the health and medical information, support, and services they need. | 1996 |  | USA |  |  | Yes |
| Your.MD | Your.MD is a digital health platform committed to helping everyone in the world find their health through self-care. Built by doctors, data scientists and digital experts, Your.MD’s aim is to help anyone, anywhere find safe and personalised information, guidance and support for their health for free. Your.MD is used by millions of people all over the world, it has the highest standards of clinical governance and backed by leading investors and health organisations. | 2012 | $17.3m | UK | 493,048 | Class 1 | Yes |

*Data Sources:*

*^1^ Provider websites*

*^2^ Crunchbase (https://www.crunchbase.com/home) Accessed: 27/01/2020*

*^3^ MHRA (https://aic.mhra.gov.uk/era/pdr.nsf/name?openpage&start=1&count=200)*

***S3 | Excluded Symptom Checkers***

***Exclusion Criteria***

The symptom checker;

- was not available for individuals to access either via the web or a dedicated application within the UK
- used another chatbot or algorithm provider as the main source
- focused on single conditions i.e. diabetes
- only focussed on paediatrics
- had narrow patient interactions where the focus was solely on condition information from an alphabetical list.

| Provider | Web / App | Reason for removal |
| --- | --- | --- |
| Patient.Info | Web/App | Uses Isabel as a base algorithm |
| FamilyDoctor.org | Web | Narrow patient interactions |
| Mayo Clinic | Web/ App | Narrow patient interactions |
| Healthline | Web | Narrow patient interactions |
| Everyday Health | Web | Uses Informedica as a base algorithm |
| Medicine.net | Web | Uses Web.MD as a base algorithm |
| Sutter Health | Web | Uses Ada Health as a base algorithm |
| RxList | Web | Uses Web.MD as a base algorithm |
| Healthychildren | Web | Paediatrics Only |
| Childmind | Web | Paediatrics Only |
| Find the right Care | Web | Uses Buoy Health as a base algorithm |
| epain assist | App | Narrow patient interactions |
| Doctor Diagnose | App | Narrow patient interactions |
| Tibot | App | Skin conditions only |
| Doctor31 | App | Does not provide advice on disposition |
| Healthdirect | Web/App | Cannot access in the UK |
| Mercy.net | Web | Use Health Navigator |
| Healthy.WA | Web | Use Healthdirect |
| HopkinsAllChildren | Web | Paediatrics Only |
| MDAnderson | Web | Cancer Only |
| Diagnosis Medical App | App | Narrow patient interactions |
| Prognosis: Your Diagnosis | App | Medic diagnosis training tool |
| Doctor on demand | App | Video consultation app |
| e-consult | Web | Not freely accessible |
| K-Health | Web/App | Cannot access in the UK |
| Cedars Sinai | Web | Use Health Navigator |

***S4 | Clinical Vignettes***

**Emergent Care - Defined as A&E now, Ambulance, appointment or advice within 1 hour**

| Vignette ID | Defined Diagnosis | Vignette Description | Simplified Symptoms | Recommended Triage Advice |
| --- | --- | --- | --- | --- |
| 1 | Acute liver failure¹ | This vignette was first published in the Semigran et al study in 2015.  They can be accessed in the appendix of the paper to be found here doi:10.1136/bmj.h3480 |  | An Ambulance should be called |
| 2 | Appendicitis¹ | This vignette was first published in the Semigran et al study in 2015.  They can be accessed in the appendix of the paper to be found here doi:10.1136/bmj.h3480 |  | The patient should attend an accident and emergency department |
| 3 | Asthma¹ | This vignette was first published in the Semigran et al study in 2015.  They can be accessed in the appendix of the paper to be found here doi:10.1136/bmj.h3480 |  | An Ambulance should be called |
| 4 | Cellulitis¹ | This vignette was first published in the Semigran et al study in 2015.  They can be accessed in the appendix of the paper to be found here doi:10.1136/bmj.h3480 |  | Emergency 1-hour appointment required |
| 5 | COPD flare (more severe)^1^ | This vignette was first published in the Semigran et al study in 2015.  They can be accessed in the appendix of the paper to be found here doi:10.1136/bmj.h3480 |  | Seeking advice from a medical professional |
| 6 | Headache due to CO poisoning | 30 yo female with mild to moderate headache. Lives with her husband who also has a mild to moderate headache that started after they got home from work. They do not have a carbon monoxide meter in the home. It is winter and they just started the wood burning stove due to cold weather. | 30 y/o f, mild / moderate headache. Partner the same, no C02 monitors | Seeking advice from a medical professional |
| 7 | Haemolytic uremic syndrome¹ | This vignette was first published in the Semigran et al study in 2015.  They can be accessed in the appendix of the paper to be found here doi:10.1136/bmj.h3480 |  | Seeking advice from a medical professional |
| 8 | Kidney stones¹ | This vignette was first published in the Semigran et al study in 2015.  They can be accessed in the appendix of the paper to be found here doi:10.1136/bmj.h3480 |  | Seeking advice from a medical professional |
| 9 | Malaria¹ | This vignette was first published in the Semigran et al study in 2015.  They can be accessed in the appendix of the paper to be found here doi:10.1136/bmj.h3480 |  | Seeking advice from a medical professional |
| 10 | Meningitis¹ | This vignette was first published in the Semigran et al study in 2015.  They can be accessed in the appendix of the paper to be found here doi:10.1136/bmj.h3480 |  | The patient should attend an accident and emergency department |
| 11 | Pneumonia¹ | This vignette was first published in the Semigran et al study in 2015.  They can be accessed in the appendix of the paper to be found here doi:10.1136/bmj.h3480 |  | Seeking advice from a medical professional |
| 12 | Pulmonary embolism¹ | This vignette was first published in the Semigran et al study in 2015.  They can be accessed in the appendix of the paper to be found here doi:10.1136/bmj.h3480 |  | The patient should attend an accident and emergency department |
| 13 | Tetanus¹ | This vignette was first published in the Semigran et al study in 2015.  They can be accessed in the appendix of the paper to be found here doi:10.1136/bmj.h3480 |  | An Ambulance should be called |
| 14 | Coronavirus | 25 yo male has fever and cough. Just returned from a work trip to China 3 days ago. | Fever, cough, abroad specifically China | Seeking advice from a medical professional |
| 15 | Heart Attack² | This vignette was first published in the Semigran et al study in 2015.  They can be accessed in the appendix of the paper to be found here doi:10.1136/bmj.h3480 |  | An Ambulance should be called |
| 16 | Stroke¹ | This vignette was first published in the Semigran et al study in 2015.  They can be accessed in the appendix of the paper to be found here doi:10.1136/bmj.h3480 |  | An Ambulance should be called |

**Urgent - Defined as appointment or seek advice >1hr up to and including 24hrs**

| Vignette ID | Defined Diagnosis | Vignette Description | Simplified Symptoms | Recommended Triage Advice |
| --- | --- | --- | --- | --- |
| 17 | Acute pharyngitis¹ | This vignette was first published in the Semigran et al study in 2015.  They can be accessed in the appendix of the paper to be found here doi:10.1136/bmj.h3480 |  | Same day appointment to see a medical professional |
| 18 | Acute pharyngitis^5^ | This vignette was first published in the Semigran et al study in 2015.  They can be accessed in the appendix of the paper to be found here doi:10.1136/bmj.h3480 |  | Same day appointment to see a medical professional |
| 19 | COPD flare (milder)¹ | This vignette was first published in the Semigran et al study in 2015.  They can be accessed in the appendix of the paper to be found here doi:10.1136/bmj.h3480 |  | Same day appointment to see a medical professional |
| 20 | Deep vein thrombosis¹ | This vignette was first published in the Semigran et al study in 2015.  They can be accessed in the appendix of the paper to be found here doi:10.1136/bmj.h3480 |  | Seeking advice from a medical professional |
| 21 | Pneumonia³ | This vignette was first published in the Semigran et al study in 2015.  They can be accessed in the appendix of the paper to be found here doi:10.1136/bmj.h3480 |  | Seeking advice from a medical professional |
| 22 | Hypertensive headache | 50 yo male with severe headache especially after waking. Has a history of high blood pressure but ran out of medication last week and stopped taking it. Has not checked his blood pressure. | Moderate / Severe headache, history high BP, medication ran out, not checked BP | Same day appointment to see a medical professional |
| 23 | Acute otitis media¹ | This vignette was first published in the Semigran et al study in 2015.  They can be accessed in the appendix of the paper to be found here doi:10.1136/bmj.h3480 |  | Seeking advice from a medical professional |
| 24 | Mononucleosis¹ | This vignette was first published in the Semigran et al study in 2015.  They can be accessed in the appendix of the paper to be found here doi:10.1136/bmj.h3480 |  | Same day appointment to see a medical professional |
| 25 | Shingles¹ | This vignette was first published in the Semigran et al study in 2015.  They can be accessed in the appendix of the paper to be found here doi:10.1136/bmj.h3480 |  | Seeking advice from a medical professional |
| 26 | Urinary tract infection¹ | This vignette was first published in the Semigran et al study in 2015.  They can be accessed in the appendix of the paper to be found here doi:10.1136/bmj.h3480 |  | Same day appointment to see a medical professional |
| 27 | Vomiting^7^ | This vignette was first published in the Semigran et al study in 2015.  They can be accessed in the appendix of the paper to be found here doi:10.1136/bmj.h3480 |  | Seeking advice from a medical professional |

**Non-Urgent - Defined as wait or seek advice more than 24 hours**

| Vignette ID | Defined Diagnosis | Vignette Description | Simplified Symptoms | Recommended Triage Advice |
| --- | --- | --- | --- | --- |
| 28 | Acute sinusitis^5^ | This vignette was first published in the Semigran et al study in 2015.  They can be accessed in the appendix of the paper to be found here doi:10.1136/bmj.h3480 |  | Routine appointment with a medical professional |
| 29 | Arthritis | 60 yo female has pain in her knee has been getting progressively worse over the past year. Difficulty kneeling, getting up from a chair and walking up stairs. Has not had any injury to the knee | 60 y/o, knee pain, getting worse, difficulty with mobility, no injury | Routine appointment with a medical professional |
| 30 | Constipation¹ | This vignette was first published in the Semigran et al study in 2015.  They can be accessed in the appendix of the paper to be found here doi:10.1136/bmj.h3480 |  | Seeking advice from a medical professional |
| 31 | Back pain^6^ | This vignette was first published in the Semigran et al study in 2015.  They can be accessed in the appendix of the paper to be found here doi:10.1136/bmj.h3480 |  | Routine appointment with a medical professional |
| 32 | Depression | 25 yo male recently married now has feelings of sadness nearly every day. No thoughts of suicide. Difficulty concentrating at work more than half the days in the past 2 weeks. Wants to just stay in bed all day. Not feeling ill. Has had some trouble sleeping several days in the past 2 weeks. | 25 yo m, feeling of sadness nearly every day, no suicide thoughts, no illness symptoms | Routine appointment with a medical professional |
| 33 | Peptic Ulcer Disease¹ | This vignette was first published in the Semigran et al study in 2015.  They can be accessed in the appendix of the paper to be found here doi:10.1136/bmj.h3480 |  | Routine appointment with a medical professional |
| 34 | Vertigo¹ | This vignette was first published in the Semigran et al study in 2015.  They can be accessed in the appendix of the paper to be found here doi:10.1136/bmj.h3480 |  | Routine appointment with a medical professional |

**Self-Care**

| Vignette ID | Defined Diagnosis | Vignette Description | Simplified Symptoms | Recommended Triage Advice |
| --- | --- | --- | --- | --- |
| 35 | Acute bronchitis¹ | This vignette was first published in the Semigran et al study in 2015.  They can be accessed in the appendix of the paper to be found here doi:10.1136/bmj.h3480 |  | Care can be self-managed at home |
| 36 | Acute bronchitis^5^ | This vignette was first published in the Semigran et al study in 2015.  They can be accessed in the appendix of the paper to be found here doi:10.1136/bmj.h3480 |  | Care can be self-managed at home |
| 37 | Acute conjunctivitis¹ | This vignette was first published in the Semigran et al study in 2015.  They can be accessed in the appendix of the paper to be found here doi:10.1136/bmj.h3480 |  | Care can be self-managed at home |
| 38 | Acute pharyngitis^5^ | This vignette was first published in the Semigran et al study in 2015.  They can be accessed in the appendix of the paper to be found here doi:10.1136/bmj.h3480 |  | Care can be self-managed at home |
| 39 | Allergic rhinitis¹ | This vignette was first published in the Semigran et al study in 2015.  They can be accessed in the appendix of the paper to be found here doi:10.1136/bmj.h3480 |  | Care can be self-managed at home |
| 40 | Back pain¹ | This vignette was first published in the Semigran et al study in 2015.  They can be accessed in the appendix of the paper to be found here doi:10.1136/bmj.h3480 |  | Care can be self-managed at home |
| 41 | Bee sting without anaphylaxis¹ | This vignette was first published in the Semigran et al study in 2015.  They can be accessed in the appendix of the paper to be found here doi:10.1136/bmj.h3480 |  | Care can be self-managed at home |
| 42 | Canker sore¹ | This vignette was first published in the Semigran et al study in 2015.  They can be accessed in the appendix of the paper to be found here doi:10.1136/bmj.h3480 |  | Care can be self-managed at home |
| 43 | Candidal yeast infection^6^ | This vignette was first published in the Semigran et al study in 2015.  They can be accessed in the appendix of the paper to be found here doi:10.1136/bmj.h3480 |  | Care can be self-managed at home |
| 44 | Eczema¹ | This vignette was first published in the Semigran et al study in 2015.  They can be accessed in the appendix of the paper to be found here doi:10.1136/bmj.h3480 |  | Care can be self-managed at home |
| 45 | Fever only | 18 yo female with fever started last night. No other symptoms. Temp is 99.6 currently and has been as high as 100.6. drinking ok, no vomiting or diarrhoea | 18 y/0 f, fever T=100.6, no other symptoms | Care can be self-managed at home |
| 46 | Influenza¹ | This vignette was first published in the Semigran et al study in 2015.  They can be accessed in the appendix of the paper to be found here doi:10.1136/bmj.h3480 |  | Care can be self-managed at home |
| 47 | Salmonella¹ | This vignette was first published in the Semigran et al study in 2015.  They can be accessed in the appendix of the paper to be found here doi:10.1136/bmj.h3480 |  | Care can be self-managed at home |
| 48 | Stye¹ | This vignette was first published in the Semigran et al study in 2015.  They can be accessed in the appendix of the paper to be found here doi:10.1136/bmj.h3480 |  | Care can be self-managed at home |
| 49 | Viral upper respiratory illness | This vignette was first published in the Semigran et al study in 2015.  They can be accessed in the appendix of the paper to be found here doi:10.1136/bmj.h3480 |  | Care can be self-managed at home |
| 50 | Viral upper respiratory illness | This vignette was first published in the Semigran et al study in 2015.  They can be accessed in the appendix of the paper to be found here doi:10.1136/bmj.h3480 |  | Care can be self-managed at home |

Table References

1. Epocrates. https://online.epocrates.com/noFrame/. 2014.

2. Lue J. NYU Medical Grand Rounds Clinical Vignette. 2012; http://www.medicine.med.nyu.edu/education/im-residency-homepage/research-opportunities/clinical-vignettes. Accessed September 8, 2014.

3. Halm EA, Teirstein AS. Clinical practice. Management of community-acquired pneumonia. N Engl J Med. Dec 19 2002;347(25):2039-2045.

4. Plantz SH, Adler JN, eds. NMS Emergency Medicine. Baltimore: Williams & Wilkins; 1998. National Medical Series for Independent Study.

5. Gidengil CA, Linder J, Beach S, Setodjian C, Hunter G, Mehrotra A. Using clinical vignettes to predict antibiotic prescribing for acute respiratory infections. In review.

6. Sirovich BE, Gottlieb DJ, Welch HG, Fisher ES. Variation in the tendency of primary care physicians to intervene. Arch Intern Med. Oct 24 2005;165(19):2252-2256.

7. Boroughs DS, Dougherty JA, Goldsmith C. Telephone Triage: Help Is Just a Call Away. http://ce.nurse.com/RVignette.aspx?TopicId=718. Accessed September 10, 2014.

***S5 Data Table | All Vignettes***

***S6 Data Table | Emergent Care Vignettes***

***S7 Data Table | Urgent Care Vignettes***

***S8 Data Table | Non-Urgent Care Vignettes***

***S9 Data Table | Self- Care Vignettes***
